# Peripheral T-cell lymphoma with hypercalcemic crisis as a primary symptom accompanied by polymyositis: A case report and review of the literature

**DOI:** 10.3892/ol.2014.2661

**Published:** 2014-11-03

**Authors:** LUYIN DING, XIBIN XIAO, LIANSHENG HUANG

**Affiliations:** Department of Hematology, The Second Affiliated Hospital of Zhejiang University Medical College, Hangzhou, Zhejiang 310009, P.R. China

**Keywords:** polymyositis, cancer-associated myositis, non-Hodgkin’s lymphoma, peripheral T-cell lymphoma, hypercalcemia

## Abstract

The present study reports the case of a 61-year-old male with polymyositis who presented with exacerbated weakness in the lower limbs and a recurrent fever that had persisted for one month. Positron emission tomography/computed tomography scans revealed multiple regions of elevated fluorodeoxyglucose metabolism in the lymph nodes, lungs, liver, spleen and bones. While symptoms of nonchalance and confusion were identified on admission, the patient’s serum calcium level was high at 3.87 mmol/l, so a hypercalcemic crisis was confirmed. A biopsy of the right lingual lymph node revealed peripheral T-cell lymphoma, not otherwise specified. The serum calcium level was restored to within the normal range following emergency measures, such as saline rehydration, diuretics, calcitonin and glucocorticoids, and partial remission was achieved following two courses of chemotherapy. The study may improve our present understanding of the diagnosis and treatment of cancer-associated myositis (CAM) and malignancy-associated hypercalcemia.

## Introduction

Polymyositis is a type of inflammatory myopathy that mostly involves the striated muscles. Typical features of polymyositis are sub-acute onset, proximal, symmetric muscle weakness, elevated serum creatine kinase levels and mononuclear cell infiltrates in the muscle biopsy. The current treatment for polymyositis includes first-line high-dose steroids and various conventional second-line treatments, such as azathioprine, cyclosporine, methotrexate, cyclophosphamide, tacrolimus, intravenous immunoglobulins, tumor necrosis factor inhibitors, rituximab and sifalimumab (1). It has been confirmed that polymyositis is highly associated with certain malignancies, particularly non-Hodgkin’s lymphomas (2). Peripheral T-cell lymphoma is a relatively rare type of non-Hodgkin’s lymphoma, and the majority of patients have a poor prognosis with frequent relapse and unfavorable outcome (3). Hypercalcemia is a common metabolic complication of these malignancies, which can present as a hypercalcemic crisis in certain patients with serum calcium levels of >3.5 mmol/l and requires emergency management (4). Common symptoms of hypercalcemia include osteoclasia, renal calculus, ectopic calcification, abdominal groans, psychiatric moans and electrocardiogram changes. Emergency management is required to restore the serum calcium level to the normal range (2.08–2.60 mmol/l) (5). The present study reports a case of peripheral T-cell lymphoma with hypercalcemic crisis as a primary symptom accompanied by polymyositis, in order to improve our present understanding of the diagnosis and treatment of such diseases.

## Case report

A 61-year-old male was admitted to The Second Affiliated Hospital of Zhejiang University Medical College (Hangzhou, Zhejiang, China) due to pain and weakness of the lower limbs that had been present for one year and a recurrent fever that had persisted for one month. One year previously, the patient had experienced pain and bilateral weakness of the muscles of the lower legs, and walking became limited. Several days later, a recurrent low to moderate fever developed and scattered red spots were visible on the extensor skin of the lower legs, with no pruritus or pain upon compression. The skin biopsy revealed erythema nodosum. The patient was administered 10 mg prednisone three times a day and the symptoms were attenuated. At 11 months prior to the present admittance, the pain and weakness of the lower limbs got worse, with involvement of the thighs and lower legs. Magnetic resonance imaging, electromyography and a biopsy of the muscle of the right thigh were performed, which diagnosed polymyositis. The condition was treated with 80 mg intravenous methylprednisolone per day and 10 mg oral methotrexate per week. Following attenuation of the symptoms, the dosage of methylprednisolone was gradually reduced.

**Figure 1:**
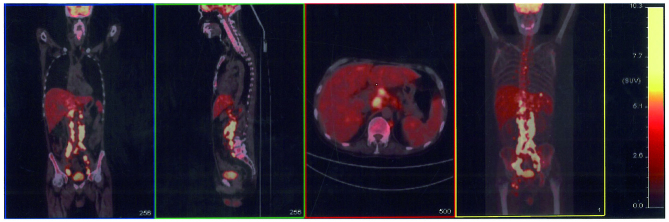
Positron emission tomography-computed tomography scan of the patient revealing multiple enlarged lymph nodes in the root of the left side of neck, mediastinum, bilateral hilus of the lungs, right cardiophrenic angle, hepatic portal area, peripancreatic area, diaphragm angle, posterior peritoneum, inter-mesangial area and anterior sacral area, and along the iliac blood vessels, bilateral iliac fossa, pelvic wall and bilateral lingual areas. The scans reveal multiple nodules in the bilateral lungs, an enlarged liver with nodular appearance of the parenchyma, an enlarged spleen with multiple low-density shadows in the parenchyma, and multiple regions of elevated fluorodeoxyglucose metabolism in the bones, such as the sternum, multiple vertebrae, and the bilateral ilia, pubis and ischia. A diagnosis of lymphoma was therefore considered.

Four months prior to admittance, the patient experienced exacerbation of the weakness in the lower limbs. A positron emission tomography/computed tomography (PET-CT) scan was performed two months later, which showed multiple regions of elevated fluorodeoxyglucose metabolism in the lymph nodes, lungs, liver, spleen and bones (Fig. 1). One month after the scan, the patient developed a high fever of 39.2°C, with no chill or cough. This raised temperature did not decrease subsequent to treatment with antibiotics (intravenous cefperazone-sulbactam, 3 g every 8 h) and, therefore, 10 mg methylprednisolone per day was administered to control the polymyositis and diclofenac potassium (50 mg, every 12 h) was administered to control the fever. No abnormal cells were found in bone marrow smears. Two lymph node puncture biopsies were performed, but the pathology showed granulomatous inflammation in each sample. Upon admission, the symptoms of nonchalance and confusion were identified, along with poor orientation and calculation abilities, and a bad memory. An enlarged lymph node could be palpated in the right lingual area. The muscles of the lower limbs were atrophied. The myodynamia of the lower limb was grade IV and the myodynamia of the upper limbs was grade V, according to Lovett’s myodynamic grading criterion (6). No other positive signs were found. A routine blood test showed a white blood cell count of 7.0×10^9^/l (normal range, 4.0–10.0 ×10^9^/l), a hemoglobin level of 94 g/l (normal range, 110–160 g/l), a platelet level of 285×10^9^/l (normal range, 100–300×10^9^/l) and a neutrophil count of 80.2% (normal range, 50–70%). The blood biochemistry results were as follows: Albumin, 23.4 g/l (normal range, 35.0–52.0 g/l); serum globulin, 45.8 g/l (normal range, 15.0–30.0 g/l); alkaline phosphatase, 1,169 U/l (normal range, 30–120 U/l); γ-glutamyl transpeptidase, 293 U/l (normal range, 9–64 U/l); aspartate aminotransferase, 59 U/l (normal level, <35 U/l); lactate dehydrogenase, 280 U/l (normal range, 140–271 U/l); calcium, 3.87 mmol/l (normal range, 2.08–2.60 mmol/l); blood urea nitrogen (BUN), 13.20 mmol/l (normal range, 2.80–7.20 mmol/l); creatine, 152 μmol/l (normal range, 53–133 μmol/l); uric acid, 749 μmol/l (normal range, 208–428 μmol/l); β2-microglobulin, 16.55 mg/l (normal range, 1.00–3.00 mg/l); and C-reactive protein, 100.9 mg/l (normal level, <6.0 mg/l). The erythrocyte sedimentation rate was 70.00 mm/h (normal level, <20 mm/h), the rheumatoid factor level was 15.8I U/ml (normal level, <15.0I U/ml), and the tests for antinuclear antibodies (ANA), anti-neutrophil cytoplasmic antibodies (ANCA), anticardiolipin antibodies and cyclic citrullinated peptide antibodies were all negative. The parathyroid hormone (PTH) level was 4.96 pg/ml (normal range, 15.00–60.00 pg/ml). A hypercalcemic crisis was diagnosed and a fluid infusion of 20 mg intravenous furosemide, 100 IU intramuscular salmon calcitonin and 10 mg intravenous dexamethasone per day was administered.

Following treatment, the patient’s memory and orientation improved, and the calcium level decreased to 2.86 mmol/l the next day. A chest CT scan showed bilateral pulmonary exudation and pneumonia was indicated, therefore, antibiotics (400 mg/day moxifloxacin and 100 mg/day fluconazole) were administered. Subsequent to the attenuation of the symptoms, a biopsy of the right lingual lymph node was performed, which showed peripheral T-cell lymphoma, not otherwise specified (NOS). The disease stage was IVB according to the Ann Arbor staging system (7), and chemotherapy consisting of 1.3 g cyclophosphamide, 60 mg liposomal doxorubicin and 40 mg vinorelbine on day 1, and 15 mg dexamethasone on days 1–5 was administered. Following two courses of chemotherapy, the result of a B-mode ultrasound and CT scan showed that the patient achieved partial remission.

## Discussion

Certain studies based on population have confirmed the association between inflammatory myopathies and malignancies. Hill *et al* (8) performed a pooled analysis of the populations in Sweden, Denmark and Finland, and revealed a strong association between dermatomyositis and malignancies [standardized incidence ratio (SIR), 3.0; 95% confidence interval (CI), 2.5–3.6], particularly ovarian (SIR, 10.5; 95% CI, 6.1–18.1), lung (SIR, 5.9; 95% CI, 3.7–9.2), pancreatic (SIR, 3.8; 95% CI, 1.6–9.0), stomach (SIR, 3.5; 95% CI, 1.7–7.3), and colorectal (SIR, 2.5; 95% CI, 1.4–4.4) cancer, and non-Hodgkin’s lymphoma (SIR, 3.6; 95% CI, 1.2–11.1). Polymyositis was associated with a higher risk of non-Hodgkin’s lymphoma (SIR, 3.7; 95% CI, 1.7–8.2) and lung (SIR, 2.8; 95% CI, 1.8–4.4) and bladder (SIR, 2.4; 95% CI, 1.3–4.7) cancer. The majority of malignancies associated with dermatomyositis are adenocarcinomas, while polymyositis is mainly associated with lymphomas (9). The majority of malignancies are discovered within one year of a confirmed diagnosis of polymyositis/dermatomyositis. It has been revealed that inflammatory myopathies are essentially a type of para-neoplastic syndrome. Additionally, the prolonged utility of immune suppressive medicine is also associated with an increased incidence of malignancies (10). The majority of patients with cancer-associated myositis (CAM) are negative for auto-antibodies and antisynthetase antibodies. In the present case, the patient developed myositis of the lower limbs one year previously, and no signs of malignancies were found at that time. Four months prior to admission, the weakness of the lower limbs was aggravated and a PET-CT scan revealed multiple lesions with enhanced metabolism around the body. Multiple biopsies were performed, which led to the final diagnosis of peripheral T-cell lymphoma, NOS. Throughout the course of the disease, the tests for auto-antibodies, such as ANA and ANCA, and antisynthetase antibodies, were negative. The clinical manifestation and disease progression matched the characteristics of CAM.

A PET/CT scan upon aggravation of the disease revealed signs of malignancy and aided in the determination of the biopsy site. The value of PET/CT and traditional examinations in polymyositis/dermatomyositis patients have been previously compared (10). Traditional examinations include chest and abdominal CT, breast molybdenum photography, gynecological examination and tests for neoplasm biomarkers. For patients of inflammatory myopathies, the positive and negative predictive values of PET/CT are 85.7 and 93.8%, respectively, whereas the positive and negative predictive values of traditional examinations are 77.8 and 95.7%, respectively. The total predictive value of PET/CT and traditional examinations is 92.7% (11). Traditional examinations expend a high amount of time and energy, while in comparison, PET/CT is effective and convenient. However, in China, PET/CT is extremely expensive and is not covered by medical insurance, therefore, consideration of economic conditions and the clinical situation is required when choosing examinations.

The primary symptom of the present patient was hypercalcemia. Common reasons for hypercalcemia are primary hyperparathyroidism and chronic renal insufficiency (during treatment with calcium tablets and vitamin D, or accompanied by tertiary hyperparathyroidism). Relatively rare reasons include vitamin D-related diseases (granulomatous diseases or vitamin D poisoning), other endocrine diseases (such as hyperthyroidism), metabolic factors (such as milk-alkali syndrome, diuretics and the utility of lithium salt) and other various reasons, including breaking limbs and familial low urinary calcium hypercalcemia (12). In the present study, the patient’s PTH level was not high and hyperparathyroidism could be ruled out. The BUN and creatine levels were moderately high, suggesting renal insufficiency, however, moderate renal insufficiency would not cause such serious hypercalcemia. Consequently, malignancy-associated hypercalcemia was diagnosed. Malignancies associated with hypercalcemia in adults include cancers such as lung cancer, head and neck neoplasms, urinary tract neoplasms and breast cancer, and also hematological malignancies such as multiple myeloma (incidence rate, 13–30%), adult T-cell leukemia/lymphoma (ATLL; 50–70%), Hodgkin’s lymphoma (5%), non-Hodgkin’s lymphoma (0.8–13%) and acute myeloid leukemia (extremely rare) (4). Among the hematological malignancies, hypercalcemia is common in multiple myeloma and ATLL, but relatively rare in non-Hodgkin’s lymphoma (13). The final diagnosis in the present study was of peripheral T-cell lymphoma with hypercalcemic crisis as a primary symptom accompanied by polymyositis; to the best of our knowledge, such a case has not previously been reported.

Hypercalcemia is caused by factors such as enhanced bone absorption, elevated calcium re-absorption by the renal tubules and elevated calcium absorption by the intestine (14). Enhanced bone absorption is the most significant cause and is mediated by the bone metastasis of tumors or by cytokines secreted by tumor cells. Common detectable cytokines include parathyroid hormone-related peptide (PTHrP), interleukin-1 (IL-1), IL-6, transforming growth factor-α, tumor necrosis factor-α, macrophage inflammatory protein-1α and calcitriol [1,25-(OH)_2_D_3_]. Occasionally, ectopic PTH secretion can be detected. However, the division of these causes into these categories is possibly too simplified and in the clinic, numerous factors could exist simultaneously to cause hypercalcemia (14). Hypercalcemia often occurs in stage III/IV cases of B-cell non-Hodgkin’s lymphoma and indicates a poor prognosis (15). Calcitriol is the most important mediator in almost all Hodgkin’s lymphomas and in 30–40% of non-Hodgkin’s lymphomas. A previous immunohistochemical study revealed that lymphoma-associated macrophages are possibly a main source of ectopic PTH (16). In the present study, the patient was stage IVB, according to the Ann Arbor staging system (5), and presented with a broad area of tumor cell infiltration. The International Prognostic Index score (17) was 5 (high-risk group), which indicated a poor prognosis. The PTHrP and calcitriol levels were not detected due to the limited time and laboratory devices in the hospital.

The main treatments for hypercalcemia include saline rehydration and dialysis, and administration of loop diuretics, bisphosphonates, calcitonin, mithramycin, gallium and corticosteroids (18). In the present case, the patient was treated with saline rehydration, furosemide, salmon calcitonin and dexamethasone, and the calcium level decreased rapidly as a consequence. The calcium concentration decreased by 1 mmol/l on the first day and was restored to within the normal range on the third day. Chemotherapy was prescribed, following which, no occurrences of hypercalcemia or renal insufficiency were noted.

In conclusion, polymyositis/dermatomyositis are strongly associated with malignancies, therefore, physicians should screen for tumors in patients with inflammatory myopathies to avoid missing the diagnosis, particularly within five years of the onset of the disease. Hypercalcemia is a common metabolic complication of malignancies, and should be considered in order to adopt appropriate treatment measures immediately. Treating the primary disease and achieving remission are fundamental methods to treat hypercalcemia. Progression has been made in the analysis of the pathogenesis of CAM, however, it is not simple or unique to a specific malignancy. Further investigations are required to elucidate the mechanisms involved.
